# Initial clinical experience with dual-layer detector spectral CT in patients with acute intracerebral haemorrhage: A single-centre pilot study

**DOI:** 10.1371/journal.pone.0186024

**Published:** 2017-11-07

**Authors:** Soo Buem Cho, Hye Jin Baek, Kyeong Hwa Ryu, Jin Il Moon, Bo Hwa Choi, Sung Eun Park, Kyungsoo Bae, Kyung Nyeo Jeon, Dong Wook Kim

**Affiliations:** 1 Department of Radiology, Gyeongsang National University School of Medicine and Gyeongsang National University Changwon Hospital, Changwon, Republic of Korea; 2 Department of Radiology, Busan Paik Hospital, Inje University College of Medicine, Busan, Republic of Korea; Institute for Bioscience and Biotechnology Research, ITALY

## Abstract

**Purpose:**

The purpose of this study was to investigate the clinical feasibility of spectral analyses using dual-layer detector spectral computed tomography (CT) in acute intracerebral haemorrhage (ICH).

**Material and methods:**

We retrospectively reviewed patients with acute ICH who underwent CT angiography on a dual-layer detector spectral CT scanner. A spectral data analysis was performed to detect contrast enhancement in or adjacent to acute ICH by using spectral image reconstructions including monoenergetic (MonoE), virtual noncontrast (VNC), and iodine overlay fusion images. We also acquired a spectral plot to assess material differentiation within lesions.

**Results:**

Among the 30 patients, the most common cause of acute ICH was chronic hypertension (18/30, 60%) followed by trauma (5/30, 16.7%), brain tumour (3/30, 10%), Moyamoya disease (2/30, 6.7%), and haemorrhagic diathesis from anticoagulation therapy (2/30, 6.7%). Of 30 patients, 13 showed suboptimal iodine suppression in the subcalvarial spaces on VNC images compared with true noncontrast images. The CT angiographic spot sign within the acute ICH was detected in four patients (4/30, 13.3%). All three tumours were metastatic and included lung cancer (n = 2) and hepatocellular carcinoma (n = 1) which showed conspicuous delineation of an enhancing tumour portion in the spectral analysis. Spectral analyses allowed the discrimination of acute haemorrhage and iodine with enhanced lesion visualization on the MonoE images obtained at lower keVs (less than 70 keV) and spectral plot.

**Conclusions:**

Even though the image quality of VNC is perceived to be inferior, it is feasible to evaluate acute ICH in clinical settings using dual-layer detector spectral CT. The MonoE images taken at lower keVs were useful for depicting contrast enhancing lesion, and spectral plot might be helpful for material differentiation in patients with acute ICH.

## Introduction

In clinical practice, computed tomography (CT) has become an essential imaging modality for the diagnosis, sequential follow-up, and assessment of treatment response of intracranial diseases. Particularly, CT is commonly performed in the field of neuroradiology as an initial screening tool to evaluate acute intracerebral haemorrhage (ICH) [1]. Although most primary ICHs originate from chronic hypertension or cerebral amyloid angiopathy, secondary ICHs are associated with intracranial vascular abnormalities (e.g., arteriovenous malformations and aneurysms), tumour, coagulopathy, or trauma [2]. Therefore, it is important to identify the underlying causes of ICH for a prediction of prognosis on the basis of neuroimaging studies for appropriate patient management. However, it may be difficult to assess the exact cause of ICH because high attenuation from acute ICH can mask true contrast enhancement or active extravasation of contrast media [3].

In many previous studies, dual-energy CT (DECT) was used to differentiate acute ICH and iodinated contrast media using the attenuation difference between two different X-ray spectra [4–7]. However, DECT with two different X-ray spectra has an inherent limitation that it does not demonstrate authentic spectral data at all energy levels of a polychromatic X-ray source. In contrast to DECT, dual-layer detector CT obtains the spectral data simultaneously during a single scan. It provides spectral data without any change to the radiologic workflow, and reduces the influence of patient motion and cross-scatter and consequently improve overall image quality. Previous studies have the use of virtual monoenergetic reconstructions to image iodine inserts using dual-layer detector spectral CT with phantoms [8, 9]. To the best of our knowledge, there is no objective study that has evaluated acute ICH on dual-layer detector spectral CT with spectral analyses using commercial software (a spectral diagnostic suite [SpDS]). Therefore, the purpose of this study was to investigate the clinical feasibility of spectral analyses using dual-layer detector spectral CT in the evaluation of acute ICH.

## Materials and methods

### Study population

The Institutional Review Board (IRB) of Gyeongsang National University Changwon Hospital approved this retrospective study and waived the need for written informed consent from the participants. Retrospective data collection and analysis were performed according to our IRB guidelines. The patient’s record and information were anonymized and de-identified prior to analysis. A review of a database at our institution identified consecutive patients with acute ICH who were admitted to the emergency department (ED) and underwent intracranial CT angiography (CTA) on a spectral CT scanner between June 2016 and October 2016. We then selected thirty-six consecutive patients with acute ICH using electronic medical records (EMRs) and a picture archiving and communicating system (PACS). We enrolled 30 of the 36 patients; six were excluded because of inadequate medical records (n = 3) or poor image quality from uncontrolled motion artefacts (n = 3). The final 30 patients who were included in this study comprised four women and 26 men (age range, 25–83 years; mean age, 59.3 years).

### Imaging acquisition and spectral data processing

CT imaging was performed on a dual-layer detector CT unit (IQon Spectral CT, Philips Healthcare, Best, The Netherlands). A true noncontrast (TNC) image and CTA in the arterial phase with spectral data were obtained for all patients. Conventional 120 kVp images and spectral images (spectral level 3) were reconstructed from the same dataset. All images were spatially registered and linked to each other in three dimensions because a single acquisition of dual-layered spectral CT was used to reconstruct both polychromatic and virtual monoenergetic (MonoE) datasets. The following acquisition parameters were applied: 120 kVp; 200 mAs; collimation, 64 × 0.625 mm; pitch factor, 0.985; rotation time, 0.5 seconds; field-of-view (FOV), 250 mm; slice thickness, 0.8 mm; slice increment, 0.4 mm; and scan time, 2.7 seconds. For the enhancement of intracranial arteries, 80 mL of contrast medium (Pamiray 370; Dongkuk Pharm., Seoul, Korea) was injected intravenously at a flow rate of 4 mL/s using a power injector (Covidien CT OptiVantage DH injector; Mallinckrodt Pharm., Dublin, Ireland). A bolus tracking method was used routinely to achieve optimal synchronization of the contrast medium flow and scanning. Once the injection is started, the bolus tracking software measures attenuation values within one internal carotid artery and a spiral scan is automatically started as soon as the threshold of 100 Hounsfield units (HU) is exceeded.

**Fig 1:**
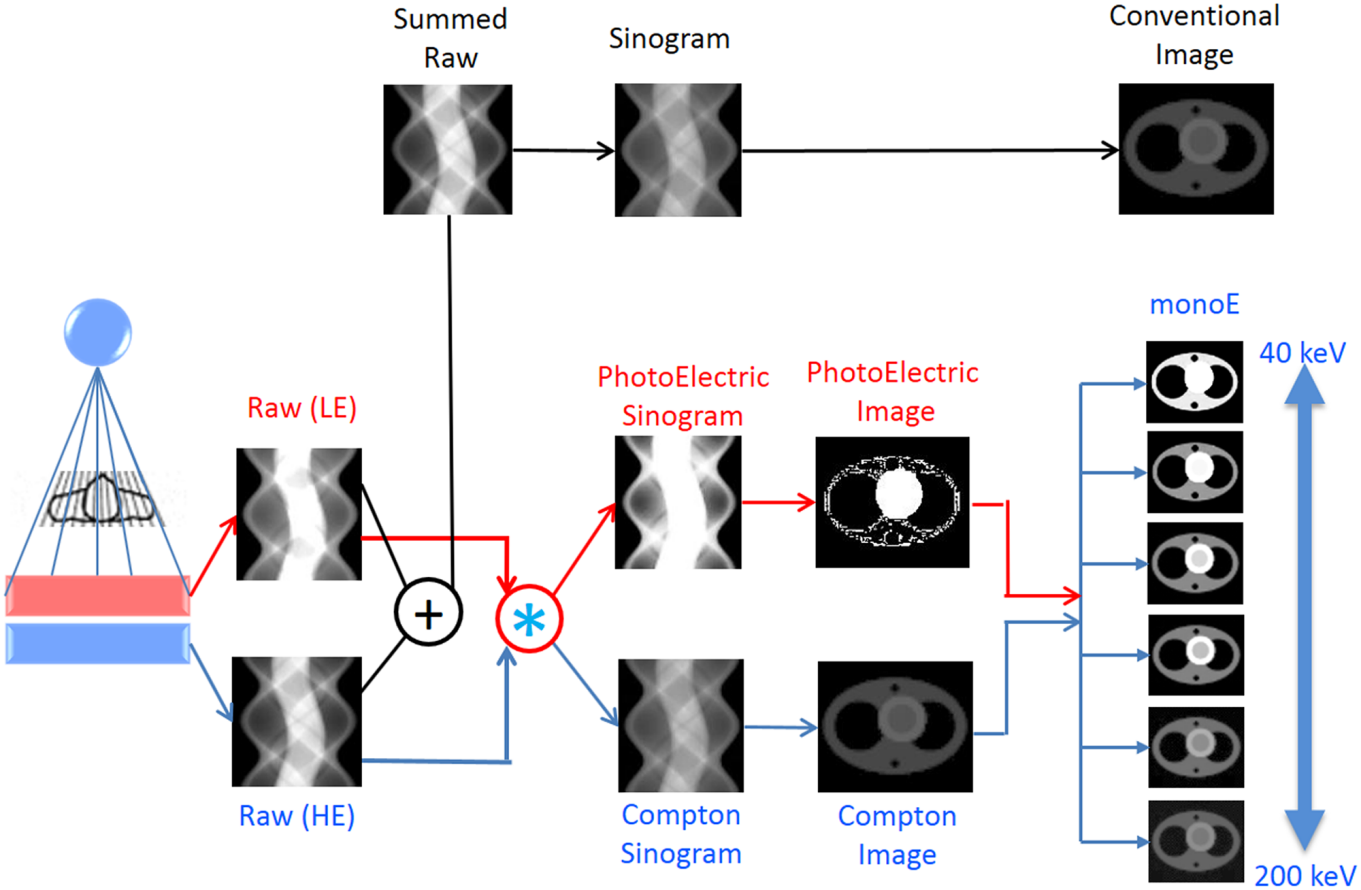
A diagram of the image acquisition and post-processing of dual-layer detector spectral CT for analysis in comparison with conventional CT.

A diagram of the data acquisition and post-processing pathway for spectral CT images is shown in Fig 1. Conventional anatomical images were generated by the summation of raw data from the lower and upper layers for all patients. Raw data from the lower and upper layers were separated into photoelectric and Compton scattering sinograms and reconstructed into a spectral base image (SBI) from which all spectral results can be derived. Spectral results are acquired within a single scan without the need for special modes because the acquisition of spectral data is dependent on the dual-layer detector rather than the X-ray tube. All spectral results were displayed in the same manner as conventional CT images using commercial SpDS software. The spectral image reconstruction for the retrospective spectral analysis included MonoE, virtual noncontrast (VNC) and iodine overlay fusion images. On MonoE images, each image series was obtained at an energy level represented by a value in the range from 40 keV to 200 keV. The appearances of MonoE images change as keV values change, even when the window and level settings are unchanged. The different appearance of the MonoE images is caused by the approximation to the iodine k-edge at 33 keV because attenuation coefficients of materials decrease as monoenergy increases to values above their k-edge energies. The pixels in these images represent HU. Principles of the material decomposition mechanism are schematically demonstrated in Fig 1 [10]. VNC images were obtained for comparison with TNC images. The iodine contrast material detected by the spectral analyses was displayed in red on iodine overlay fusion images that were obtained from arterial-phase images. Volume-rendered (VR) and maximum intensity projection (MIP) images of the intracranial arteries were obtained using a bone removal method.

### Imaging analyses

An attending neuroradiologist (H.J.B. with 7 years of experience in brain, head, and neck imaging) interpreted the images while blinded to the clinical history of each patient. For all patients, the initial assessment was performed using conventional anatomical CT images. Then, an additional spectral analysis was performed to detect contrast enhancement in or adjacent to the acute ICH. All spectral data acquired during the scan were easily accessed from the PACS for the retrospective spectral data analysis. To compare with TNC images, we evaluated VNC images, and image quality of VNC images was graded according to the following items by visual assessment: (a) differentiation of grey-white matter at the level of the lateral ventricles; (b) demarcation of basal ganglia at the level of foramina of Monro; (c) demarcation of the cerebral sulci; and (d) visualization of intracranial vessels. For each of these items, the dichotomization simplified the image quality assessments to inadequate versus sufficient. In addition, we analysed spectral plots to evaluate material differentiation (S1 File). To acquire a spectral plot, the same neuroradiologist manually drew region of interest (ROI) defining the enhancing tumour portion or active extravasation on a selected representative MonoE image while avoiding traversing vessels and tumour necrosis. In addition, intracranial arteries were reviewed on VR and MIP images to evaluate the vascular cause of the acute ICH.

## Results

### Study population

**Fig 2:**
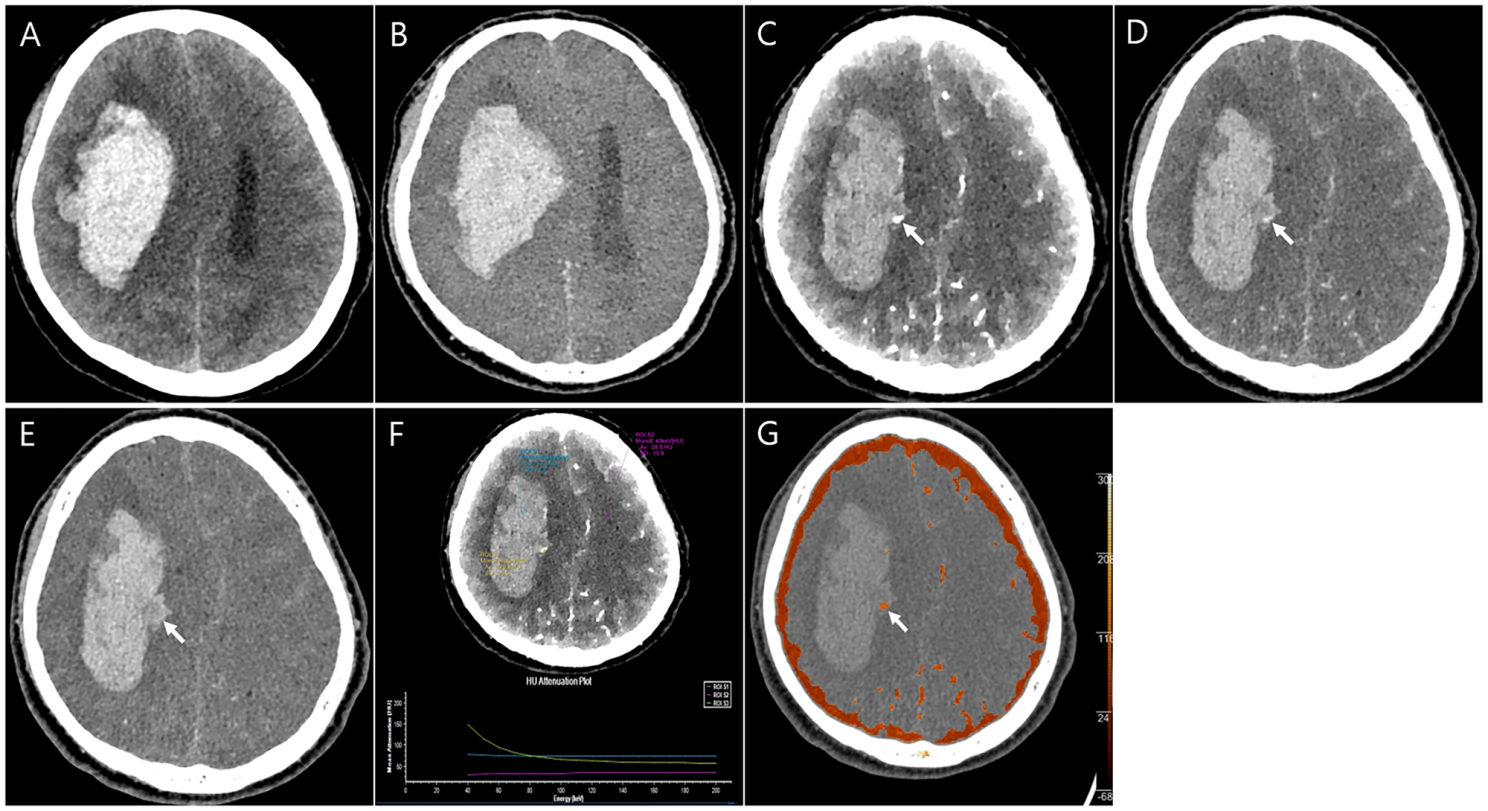
A 48-year-old man with Moyamoya disease. (A). A true noncontrast (TNC) image shows a large lobar haematoma in the right fronto-parietal lobe. (B). A virtual noncontrast image clearly shows a haematoma with image quality similar to the TNC image. (C–E). Monoenergetic (MonoE) images at 40 keV (C), 80 keV (D), and 140 keV (E). A small, dot-like enhancement (arrow) is noted in the medial aspect of the haematoma, suggesting active extravasation at 40 keV, and this lesion has similar attenuation to the haematoma as the keV values increase. (F). On a spectral plot from a MonoE image at 40 keV, active extravasation reveals a gradual decrease in HU values with a steep slope (ROI 3), suggesting iodine. (G). An iodine overlay fusion image based on 80 keV MonoE image also shows conspicuous delineation of a small, active extravasation (arrow).

**Fig 3:**
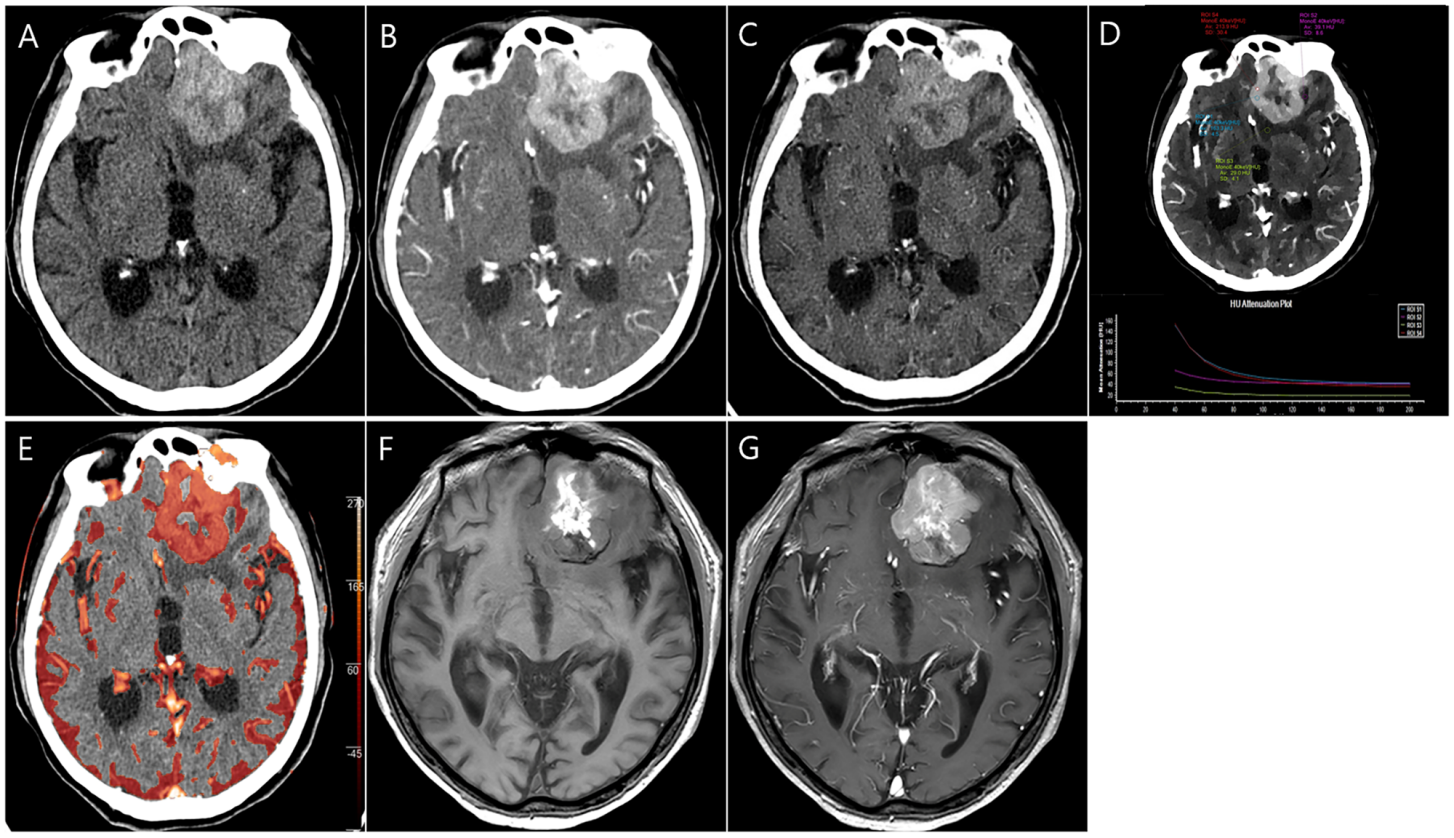
An 82-year-old man with lung cancer. (A). A true noncontrast (TNC) image shows a lobular haematoma in the left frontal lobe. (B). An enhanced image also demonstrates strong hyperattenuation but does not provide exact information regarding the enhancing component. (C). A virtual noncontrast image clearly shows a haematoma; however, the image is slightly smoothed and there is incomplete suppression of the intracranial arteries compared with the TNC image. (D). On a spectral plot from a MonoE image at 40 keV, peripheral hyperattenuation shows a gradual decrease in HU values with a steep slope (ROI 1) similar to a tumour vessel (ROI 4), suggesting a contrast-enhancing tumour portion. Two hypoattenuation foci (ROI 2, 3) show constant HU values regardless of the keV value. (E). An iodine overlay fusion image based on 80 keV MonoE image shows the left frontal lesion in red, representing the iodine component. (F, G). A non-enhanced axial T_1_-weighted image (F) shows central hyperintensity with peripheral isointensity in the left frontal lesion. An enhanced T_1_-weighted image (G) demonstrates strong peripheral enhancement of the lesion, suggesting an underlying brain tumour. This tumour was pathologically confirmed as a haemorrhagic metastasis from lung cancer (adenocarcinoma) by tumorectomy.

The clinical data of the study patients are summarized in Table 1. CTA was obtained successfully for all patients without any side effect or complication. In the 30 patients included in the study, various neurologic symptoms were noted, including headache (n = 12, 40%), abrupt mental status change (n = 8, 26.7%), motor weakness (n = 6, 20%), sensory change (n = 3, 10%), and dizziness or vertigo (n = 1, 3.3%). The locations of ICH were as follows: the thalamus (10/30, 33.3%); basal ganglia (9/30, 30%); lobar pattern (7/30, 23.3%); pons (2/30, 6.7%); and cerebellum (2/30, 6.7%). The most common cause of acute ICH was chronic hypertension (18/30, 60%) followed by trauma (5/30, 16.7%), brain tumour (3/30, 10%), Moyamoya disease (2/30, 6.7%), and haemorrhagic diathesis by anticoagulation therapy (2/30, 6.7%). A diagnosis of hypertensive ICH or haemorrhagic diathesis-related ICH was based on the clinical information and resolution of the lesions on follow-up CT or magnetic resonance (MR) imaging. The CT angiographic spot sign within the ICH was detected in four patients (13.3%, Fig 2). All three tumour cases were diagnosed as metastatic tumours from lung cancer (n = 2) or hepatocellular carcinoma (n = 1) on the basis of the clinical background and additional or follow-up imaging, including CT, MR, and positron emission tomography (PET)-CT imaging. The ICHs from metastatic tumours showed a vividly enhancing solid tumour portion on spectral CT images (Fig 3).

**Table 1:** Clinical characteristics of patient population.

Characteristics	
Age (years)	59.3 ± 13.7
Gender	Female: Male = 4 (13.3): 26 (86.7)
Location of ICH	
Lobar	7 (23.3)
Basal ganglia	9 (30)
Thalamus	10 (33.3)
Pons	2 (6.7)
Cerebellum	2 (6.7)
Cause of ICH	
Hypertension	18 (60)
Trauma	5 (16.7)
Moyamoya disease	2 (6.7)
Tumour	3 (10)
Haemorrhagic diathesis	2 (6.7)
Spot sign	
Presence	4 (13.3)
Absence	26 (86.7)

Note.—Data presented in parentheses are percentage of each item. AVM, arteriovenous malformation; ICH, intracerebral haemorrhage.

### Image analyses

Of these 30 patients, the results of dichotomized assessment regarding to VNC images as follows: (a) differentiation of grey-white matter at the level of the lateral ventricles; inadequate (7, 23.3%) vs. sufficient (23, 76.7%), (b) demarcation of basal ganglia at the level of foramina of Monro; inadequate (9, 30%) vs. sufficient (21, 70%), (c) demarcation of the cerebral sulci; inadequate (12, 40%) vs. sufficient (18, 60%) and (d) visualization of intracranial vessels; inadequate (13, 43.3%) vs. sufficient (17, 56.7%). Twelve cases in the item (c) revealed inconspicuous delineation of cerebral sulci, which reduced overall image quality and interpretability. According to item (d), 13 inadequate cases showed suboptimal iodine suppression in the subcalvarial spaces on VNC images, which can make distinguishing blood vessels from extraaxial haemorrhages difficult. However, these blood vessels were easily identified during assessment because of their serpentine continuity along the predictable vascular course.

In the analyses of MonoE images, they tended to enhance visualization of the contrast-enhanced tumour portion or active contrast extravasation at low keV values and reduced beam hardening at high keV values; thus, the conspicuity of the lesions was increased. In particular, use of MonoE images acquired at keV values less than 70 keV allowed better visualization of contrast-enhancing structures such as blood vessels, contrast extravasation, or enhancement of the tumour portion on the visual assessment. We also found that overall image noise increased as keV of MonoE image decreased. In addition, spectral plots allowed discrimination between haemorrhage and iodine in patients with brain tumours and active extravasation. On the spectral plots, iodine showed the highest HU values on 40 keV MonoE images and decreased HU values with a steep slope on higher keV MonoE images. However, the haemorrhage and brain parenchyma demonstrated low HU values with an unchanged slope (Figs 2 and 3). At 40 KeV, iodine has more than a two-fold higher HU value compared to that at 120 keV. At higher values above 100 keV, all materials such as iodine, cerebrospinal fluid, the brain parenchyma, and haemorrhage had similar HU values. In addition, the intracranial arteries also became more obvious with the effective suppression of bone beam hardening by the skull vault on MonoE images at higher keV values (100–120 keV) during the spectral analysis.

## Discussion

The overall worldwide incidence of ICH ranges from 10 to 20 cases per 100,000 in the population and increases with age [2, 9]. ICH can be classified as either primary or secondary depending on the underlying aetiology of the bleeding [1, 2]. Therefore, it is important to evaluate the underlying causes of ICH for appropriate patient management. Although unenhanced CT is the standard imaging modality for the detection of ICH in clinical practice, contrast-enhanced CT, CTA, and MR imaging may be needed to further detect information regarding ICH [1, 11, 12]. It is well known that the presence of active contrast extravasation within an ICH on CTA has been associated with haematoma expansion and patient mortality [11, 12]. In addition, approximately 10% of ICH occurs from tumour bleeding, and it can be difficult to detect tumour enhancement because of the masking effect of the coexisting high attenuation from acute ICH [3].

In a previous study, DECT was used to differentiate high ICH attenuation from that of iodine by the attenuation difference between the two materials at different energies, which is based on the photoelectric effect and Compton scattering. While the photoelectric effect is strongly energy-dependent, Compton scattering is not. CT attenuation depends on the photon’s energy, the atomic number, and the concentration of the material being scanned. Therefore, materials in the body can be differentiated by using different X-ray spectra, an effect that is particularly useful for materials with a large atomic number because of the photoelectric effect. Iodine has a high atomic number and shows a large attenuation difference between high- and low-energy X-rays, whereas haematomas do not. This was the basic mechanism used to differentiate iodine from acute ICH in previous studies using DECT [4–6].

However, to the best of our knowledge, there is no study that evaluated ICH using dual-layer detector spectral CT with spectral data analyses. In this study, we were able to investigate the clinical feasibility of dual-layer detector spectral CT for evaluating acute ICH. This technique is the most up-to-date commercial technical approach, which is based on an energy-resolving layer detector with a single polychromatic spectrum. In the dual-layer detector, the sensitivities of the two detectors are determined by the scintillator materials such as ZnSe or CsI in the upper layer and Gd_2_ O_2_S in the lower layer, which are related to the spectral resolution. In this model, spectral data were reconstructed at different virtual monoenergies, resulting in virtual MonoE images. These MonoE images reflect the HU values of the scanned region at one specific energy, in contrast to the HU values derived from a conventional polychromatic spectrum.

In the present study, all three brain tumours were correctly diagnosed based on the spectral analysis, and CTA spot signs suggesting active bleeding were more easily detected with increased conspicuity in the spectral analysis, especially on MonoE images taken at lower keV values (< 70 keV) than on conventional images. In addition, spectral plots showed a simple visualization of material decomposition in patients with brain tumours and active extravasation. The result of the spectral plot is based on MonoE images and the spectral plot represents a change in HU for the ROI according to the change in keV. Therefore, the slope of the ROI in the spectral plot reveals material composition because the X-axis represents keV and the Y-axis represents HU. We suggest that the spectral plot can be a time-efficient analysis to differentiate materials without reviewing all MonoE images according to the predefined keV levels. Similar to the material characterization capabilities of DECT that have been previously studied [6, 11, 12], in this pilot study, we found that it is possible to differentiate ICH from coexisting iodinated contrast materials using spectral CT with a dual-layer detector. However, in this study, all CT images were evaluated by a single reader, and visual qualitative assessment was done with a small proportion of tumour and active bleeding. These factors may have affected the results of the current study in terms of objectivity. Therefore, additional studies are needed to clarify these issues.

Although we used visual assessment for image analyses, we also found that overall image noise increased as MonoE keV decreased which was documented in the previous studies [13, 14]. This is an inverse relation between improved visualization of the contrast enhancing structures and increased overall image noise at low keV, which can have an adverse effect on the image interpretations. However, this issue may be overcome by changing the keV value, i.e., MonoE images should be taken at 40 or 50 keV for evaluation of blood vessels or contrast-enhancing lesions, and higher keV MonoE or polychromatic images should be used for evaluation of other structures of interest.

In the current study, bone beam hardening artefacts were reduced in images taken at higher keV values. Therefore, it may provide additional information about intracranial lesions at the level of the skull base or intraosseous segments of the intradural and extradural arteries than does conventional CTA. In addition, CTA with dual-layer detector spectral CT provided anatomical information as well as the ability to characterize structures based on material content within a single scan. Although image post-processing with DECT is only conducted at a workstation, spectral data can be accessed easily from a PACS for retrospective, on-demand, spectral analysis to further investigate a particular ROI. Because of this benefit, there is no need to recall the patient for additional imaging in daily clinical practice. Therefore, we believe that these advantages of dual-layer detector spectral CT can be helpful in determining a time-efficient diagnosis in a clinical setting, especially pediatric, non-cooperative, and emergent patients.

Although we assessed overall image quality of VNC and TNC images using dichotomized scoring, the results were not sufficient to validate the image quality of VNC images as an alternative to TNC images owing to the small sample size and visual assessment by a single radiologist. In this pilot study, suboptimal image quality of VNC images was observed in 13 patients (43.3%) because of insufficient iodine suppression of leptomeningeal or bridging vessels in the subcalvarial spaces, which can mimic extraaxial haemorrhages in the acute trauma setting. While suboptimal iodine suppression usually occurred in the subcalvarial blood vessels, these were readily recognizable and were not confused with pathological condition because of their serpentine continuity along the predictable vascular course. The suboptimal demarcation of the cerebral sulci was another drawback of VNC images, which reduced overall image quality. This issue can decrease the accuracy during image interpretation in the clinical practice and may necessitate additional TNC images to confirm the diagnosis of subcalvarial pathologies such as subarachnoid haemorrhage or cortical microhaemorrhage. Further, additional studies having larger sample sizes and involving quantitative image assessment should be carried out in the near future to validate our results.

In this study, the radiation dose from a single spectral CT scan is approximately 1.2 mSv, which is lower than that from a typical conventional CTA scan and similar to that of a single set of DECT-CTA images [11, 15, 16]. The average radiation dose in this study was about 2.3 mSv because we routinely acquired TNC images for comparison with VNC images. Unfortunately, we could not show that TNC images were replaced with VNC images to reduce the spectral CT radiation dose. Therefore, we expect that CTA with spectral CT can be an ideal imaging modality for patients with acute ICH as it reduces both scanning time and radiation dose in addition to achieving further improvement of VNC image quality.

There are several limitations to this study. First, there was an unavoidable selection bias because of the study’s retrospective nature. Second, a relatively small number of patients were included in this pilot study with a high proportion of hypertensive ICH. It may be influenced by the particular situation regarding to the early period of the official opening of our hospital. Therefore, our study had a weakness for generalization. Third, we did not investigate diagnostic accuracy by statistical analysis and perform a quantitative assessment because this pilot study was focused on early clinical experience with dual-layer detector spectral CT. Lastly, we used the image review of only one attending neuroradiologist. Therefore, further studies with larger sample sizes, various ICH aetiologies and multi-reader assessment of image quality are required for the validation of our results.

## Conclusions

In conclusion, it is feasible to evaluate acute ICH in the clinical settings using dual-layer detector spectral CT even though the image quality of VNC images is perceived to be inferior yet. In this study, the MonoE images taken at lower keVs were beneficial for depicting contrast enhancement; further, spectral plot might be helpful for material differentiation in patients with acute ICH. Spectral data analyses offer additional diagnostic information based on material decomposition with a time-efficient workflow because the appearance of the synthesized MonoE images changed as the predefined keV values changed. We expect that further research may reveal additional applications for dual-layer detector spectral CT and also validate its clinical performance.

## Supporting information

S1 File This is the minimized data set regarding to imaging analyses of this study. (XLSX)
